# Effects of corn syrup solids containing maltobionic acid (maltobionic acid calcium salt) on bone resorption in healthy Japanese adult women: A randomized double‐blind placebo‐controlled crossover study

**DOI:** 10.1002/fsn3.1387

**Published:** 2020-01-09

**Authors:** Daiki Suehiro, Ayaka Nishio, Junya Kawai, Ken Fukami, Motoko Ohnishi

**Affiliations:** ^1^ San‐ei Sucrochemical Co., Ltd. Chita Japan; ^2^ Graduate School of Bioscience and Biotechnology Chubu University Kasugai Japan; ^3^ College of Bioscience and Biotechnology Chubu University Kasugai Japan

**Keywords:** bone metabolism, bone resorption, DPD, maltobionic acid, u‐NTx

## Abstract

Maltobionic acid is known to have an inhibitory effect on the differentiation of osteoclasts, and it has also been reported in an intervention trial that ingestion of corn syrup solids containing maltobionic acid maintained and increased the bone density of postmenopausal women. However, there is no information on whether maltobionic acid improves bone metabolism in humans. Therefore, we evaluated the influence of corn syrup solids containing maltobionic acid (maltobionic acid calcium salt) on bone resorption markers in healthy Japanese women. Forty‐one individuals were selected from 68 participants and assigned to two groups: 21 individuals in the test food antecedent group and 20 individuals in the placebo food antecedent group; individuals in the first group ingested 4 g of corn syrup solids containing maltobionic acid, and subjects in the second group ingested 4 g of placebo (hydrous crystalline maltose and calcium carbonate) for 4 weeks. Bone resorption marker levels (DPD and u‐NTx) were evaluated by urinalysis. Forty subjects completed the study, and no adverse events related to the test food were observed. Fourteen subjects were excluded prior to the efficacy analysis because of conflict with the control criteria; the remaining 33 subjects were analyzed. Consumption of corn syrup solids containing maltobionic acid was maintained; DPD and u‐NTx values were improved (*p* < .05). These results indicate that corn syrup solids containing maltobionic acid might contribute to suppress bone resorption and improve bone metabolism in postmenstrual women. (UMIN‐CTR ID: UMIN000034257; Foundation: San‐ei Sucrochemical Co., Ltd.).

## Graphical abstract

Changes in bone resorption markers (DPDs, u‐NTx) before and after the ingestion study. The postintervention values of DPD and u‐NTx did not differ in the placebo food group compared to the preintervention values; however, these values reduced significantly in the test food group.

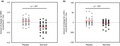

## 1 INTRODUCTION

According to the NIH, osteoporosis is “A disease characterized by low bone mass and microarchitectural deterioration of bone tissue, leading to enhanced bone fragility and a consequent increase in fracture risk” (NIH Consensus Development Panel, 2001). In the ultra‐aging society of Japan, the number of patients with osteoporosis has been increasing year by year with current estimates suggesting that 12.8 million Japanese citizens over 40 years old are affected, 70% of whom are women (Yoshimura et al., 2010, 2009). Bone density peaks at puberty in both sexes, and decreases with age thereafter (Carrié Fässler & Bonjour, 1995; Ilich, Badenhop, & Matkovic, 1996). Women, in particular, are known to experience a sharp decrease in bone mass after menopause (Orito, Kuroda, Onoe, Sato, & Ohta, 2009).

Bone is a tissue composed mainly of hydroxyapatite, a type of calcium phosphate, and type I collagen. Within the bone, old bone is lysed and resorbed by osteoclasts (bone resorption) and new bone is formed by osteoblasts (osteogenesis) in a continuous cycle (Frost, 1964; Parfitt, 1994). It has been reported that an imbalance in bone metabolism, where an increase in bone resorption by osteoclasts overtakes bone formation, leads not only to a decrease in bone density and an increased risk of osteoporosis, but also drives the risk of additional diseases, such as rheumatoid arthritis (Haugeberg, Uhlig, Falch, Halse, & Kvien, 2000) and periodontitis (Mohammad, Hooper, Vermilyea, Mariotti, & Preshaw, 2003; Penoni et al., 2016). Estrogen deficiency, which also occurs frequently in postmenopausal women, induces osteoclast activation (Zebaze et al., 2010). Therefore, in addition to keeping the bone healthy, suppression of excessive bone resorption is important for maintaining quality of life (QOL) (Silverman, Minshall, Shen, Harper, & Xie, 2001).

**Figure 1:**
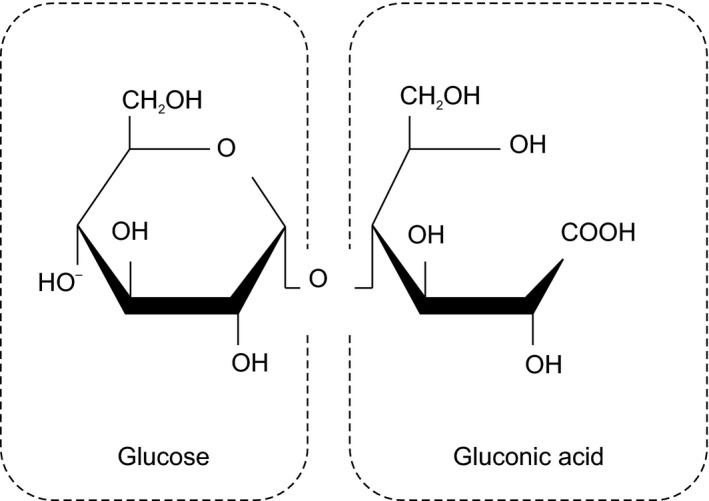
Structure of maltobionic acid

Maltobionic acid, a disaccharide in which glucose is α‐1,4‐bonded to gluconic acid, is a component of honey that has been a part of the human diet since ancient times (Figure 1). Although it is a saccharide, it has a mildly sour taste and high water solubility despite ion binding with calcium, a feature that leads to a stable salt formation with inorganic cations (Suehiro, Okada, Fukami, Otuka, Nakagawa, & Hayakawa, 2017). Previously, we have reported using rat models that maltobionic acid enhances calcium and magnesium absorption by maintaining the solubilized state of minerals throughout the intestinal tract and increasing the amount of calcium in the femur (Suehiro et al., 2017; Suehiro, Okada, Fukami, Nakagawa, & Hayakawa, 2019). In vitro studies with cultured cells confirmed that maltobionic acid has an inhibitory effect on the differentiation of osteoclasts (manuscript in preparation). In addition, a 24‐week intervention trial revealed that the ingestion of corn syrup solids containing maltobionic acid (maltobionic acid calcium salt) maintained and increased the bone density in postmenopausal women (Fukami, Suehiro, & Takara, 2019). However, there is no information on whether maltobionic acid improves bone metabolism in human clinical trials. Therefore, this study has investigated the effect on bone resorption markers in Japanese adult women up on ingesting maltobionic acid calcium salt.

## 2 SUBJECTS AND METHODS

### 2.1 Study design and participants

This was a randomized, double‐blind, placebo‐controlled, crossover trial. The study participants were publicly recruited and included Japanese adult women who worked at Chubu University (Aichi, Japan). A preliminary questionnaire was administered to those who gave written informed consent confirming their wish to participate in the study. Sixty‐eight women aged 40–69 years were enrolled in the study. The following exclusion criteria were used to select the final study participants by excluding those with: (a) a medical history of osteoporosis, malignant tumor, heart failure, or myocardial infarction; (b) the presence of other diseases (osteoporosis, arrhythmia, liver dysfunction, kidney dysfunction, cerebrovascular disease, rheumatism, diabetes, dyslipidemia, hypertension, or other chronic disease); (c) the regular use of pharmaceuticals (including Kampo) or supplements; (d) the regular ingestion of foods for specialized health use or with functional claims; (e) weekly ingestion of calcium, vitamin D, vitamin K, magnesium, isoflavones (including daidzein, genistein, equal) and all other supplements, foods for specialized health use, foods with functional claims, and foods with nutritional function claims that could affect bone metabolism; and (f) allergies (pharmaceuticals and foods related to the test foods in this study). The subjects in their premenopausal period or those who had undergone premature menopause due to genetics, illness, or medical procedures were also excluded.

This study's protocol received approval from the Chubu University Certified Review Board on September 21, 2018 (no. 300019‐2). The study was conducted with full consideration of medical ethics and in accordance with the Declaration of Helsinki (2013) and the Ethical Guidelines for Medical and Health Research Involving Human Subjects. Testing was mainly conducted by the Chubu University. This study was registered with the University Hospital Medical Information Network (no. UMIN000034257). The number of subjects and intervention period included in the study was based on bone resorption markers in reference to the literature by Mori, Sagara, Ikeda, Miki, and Yamori (2004) and studies by Uesugi, Fukui, and Yamori (2002).

### 2.2 Selection, randomization, and blinding

Forty‐one postmenopausal women who had undergone natural menopause were selected from 68 participants and assigned to two groups: 21 individuals in the test food antecedent group and 20 individuals in the placebo food antecedent group, where those in each group did not differ greatly in age or BMI. Group allotments were conducted by an intermediary study controller using StatLight #11 (Yukms Co., Ltd.). The group allotments were unknown to study participants, principal investigator, outcome assessors, and all other staff involved in this study; none of these individuals were involved in the allotment process.

### 2.3 Test food

The test food used in the intervention was corn syrup solids containing maltobionic acid (SourOligo C, San‐Ei Sucrochemical Co., Ltd.) packaged in stick‐shaped packet form (4 g/packet). Corn syrup solids containing maltobionic acid consisted of 5.3% solids, 4.4% calcium, 60.3% maltobionic acid, 16.7% maltotrionic acid, and 13.3% other carbohydrates. The placebo food was a mix of 89.0% crystalline maltose hydrate (Hayashibara Co., Ltd.) and 11.0% calcium carbonate (Sankyo Seifun Co., Ltd.) contained in stick‐shaped packets (4 g/packet). Its calcium component was 176 mg out of 4 g, the same as the test food. Both foods were in a powdered state. Prior to the start of the study, it was confirmed that the foods could not be distinguished based on odor or color.

### 2.4 Outcome measures

Examinations were performed a total of four times before and after the intervention.

1. Primary outcome: Urinary bone resorption marker

Approximately 10 ml of first morning urine was collected from test participants. Two bone turnover markers, urinary deoxypyridinoline (DPD) and urinary type I collagen‐cross‐linked‐N‐telopeptide (u‐NTx), were measured and corrected by urinary creatinine content. DPD and u‐NTX were measured using the ELISA kits Osteolinks‐DPD (SB Bioscience Co., Ltd.) and OSTEOMARK (Alere Medical Co., Ltd.), respectively. Urinary creatinine was measured using LabAssay Creatinine (FUJIFILM Wako Pure Chemical Corporation).

- Urinalysis

Protein (Pro), glucose (Glu), urobilinogen (Uro), bilirubin (Bil), ketone bodies (Ket), pH, and occult blood (Bld) were measured from first morning urine. UROPAPER III EIKEN and US‐3100R (EIKEN CHEMICAL Co., Ltd.) were used for measurements.

- Diet survey

Calcium (Xu, McElduff, D'Este, & Attia, 2004), potassium (Macdonald et al., 2008), vitamin D (Matsumoto et al., 2005), and vitamin K (Feskanich et al., 1999) are known to maintain bone density and bone metabolism; the test foods in this study are expected to have similar effects. To correctly assess the effects of the test foods, a diet survey was performed using a brief‐type self‐administered diet history questionnaire (BDHQ) before and after the interventions began. The BDHQ questionnaire—based on a computer algorithm that takes into account the energetics and selected nutrients consumed to estimate the intake—was used to investigate the ingestion of 56 foods and beverages over the last month (Kobayashi et al., 2012, 2011).

### 2.5 Statistical analysis

All outcomes were presented as mean ± *SD*. The primary outcome, DPD, and u‐NTX, were tested for carryover and aging effects, thereby confirming that the crossover design was adequate. Results were examined in intragroup and intergroup comparisons. Intragroup comparisons were made by paired Student's *t* tests between pre‐ and postintervention measures. Intergroup comparisons were made by comparing measurements and changes at individual time points between the test food and placebo food groups. Changes were obtained by subtracting preintervention measurements from postintervention measurements. Preintervention measures and changes were compared between groups using Student's *t* tests and postintervention measures using ANCOVA with preintake measures as covariates. All statistical analyses were performed using two‐sided testing, and the standard of significance was set at 5%. The software used was Microsoft Excel 2010 and Bell Curve for Excel (Social Survey Research Information Co., Ltd.). Redundancy with other time points or other items was given no consideration.

## 3 RESULTS

### 3.1 Analysis of subjects

**Figure 2:**
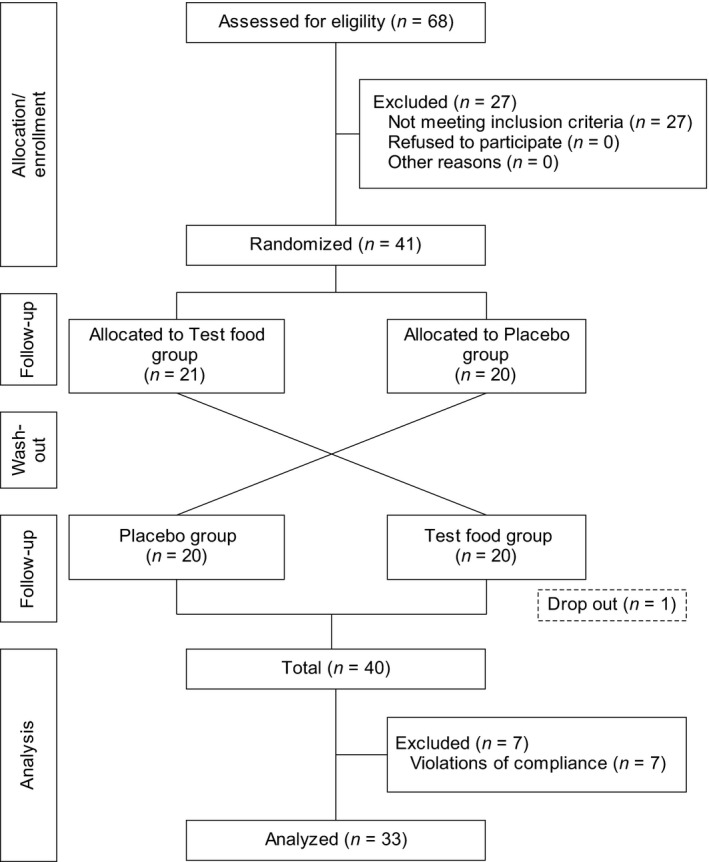
Follow‐up chart for the study participants

Figure 2 shows a follow‐up flowchart for the study participants. Of the 68 individuals who consented to participate in this study, 27 were excluded during interviews with the principal investigator or because of the inclusion/exclusion criteria. Finally, 41 individuals were enrolled in this study and assigned to the test food antecedent group (21 individuals) and the placebo food antecedent group (20 individuals). We excluded one dropout from the study and seven violators of compliance (unsubmitted diaries, ingested food consumption rates of 80% or less, and inadequacies at the time of urine sample submission). Though the study participants whose urinary bone resorption markers (DPD and u‐NTx) in the urinalysis before and after the intervention deviated from the standard values specified in the guideline (Nishizawa et al., 2019) were to be excluded, none of the participants were eligible. Therefore, the object of the analysis was to carry out the analysis in the “per protocol set,” which amounts to 33 subjects (mean age, 52.3 ± 5.3 years).

### 3.2 Efficacy assessment

**Figure 3:**
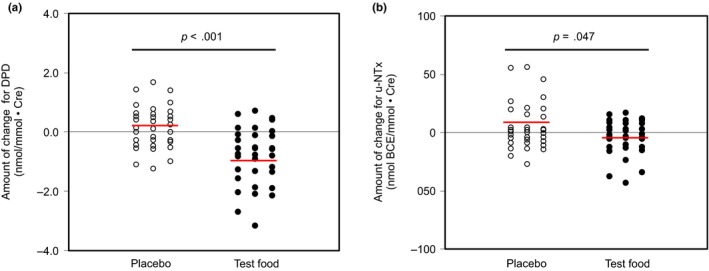
Changes in (a) DPD and (b) u‐NTx

The background characteristics of the 33 subjects in the subject population for the efficacy analysis are shown in Table 1. The efficacy assessment items (DPD and u‐NTx) are shown in Table 2 and Figure 3. Urinalysis results are shown in Table 3. The results of the dietary survey by BDHQ are shown in Table 4.

1. Primary outcome: Urinary bone resorption marker

**Table 1:** Characteristics of subjects

	The entirety (*n* = 41)	Analytical subject population (*n* = 33)
Age	54.8 ± 5.1	55.1 ± 4.8
Body height (cm)	157.8 ± 5.0	157.5 ± 5.1
Body weight (kg)	52.4 ± 6.4	53.8 ± 6.5
BMI (kg/m^2^)	21.1 ± 2.5	21.3 ± 2.4

Note

Values are mean ± *SD*.

**Table 2:** Changes in bone metabolism markers

Marker	Unit	Group	*n*	Preingestion	Post‐4W	Preingestion versus Post‐4W
						Amount of change
DPD	nmol/mmol·Cr	Test food	33	4.81 ± 1.37	4.01 ± 1.11	−0.81 ± 0.96*, **
		Placebo		4.61 ± 0.98	4.80 ± 1.08	0.19 ± 0.71
u‐NTx	nmol BCE/mmol·Cr	Test food	33	46.4 ± 17.9	42.9 ± 18.1	−3.5 ± 15.0**
		Placebo		47.9 ± 19.9	53.5 ± 28.7	5.6 ± 20.0

Note

Values are mean ± *SD*.

* *p* < .05 (vs. Preingestion).

** *p* < .05 (vs. Placebo).

**Table 3:** Changes in urinalysis parameters

Parameter	Reference value	Group	*N*	Preingestion	Post‐4W
Protein	(−)	Test food	33	(−):33	(−):31, (±):2
		Placebo	33	(−):33	(−):31, (±):2
Glucose	(−)	Test food	33	(−):33	(−):33
		Placebo	33	(−):33	(−):33
Urobilinogen	(±)	Test food	33	(±):33	(±):33
		Placebo	33	(±):33	(±):33
Bilirubin	(−)	Test food	33	(−):33	(−):33
		Placebo	33	(−):33	(−):33
Ketone	(−)	Test food	33	(−):32, (+):1	(−):32, (+):1
		Placebo	33	(−):33	(−):32, (+):1
pH	5.0–7.5	Test food	33	(5.0–7.5):32, (8.0):1	(5.0–7.5):31, (8.0):2
		Placebo	33	(5.0–7.5):30, (8.0):3	(5.0–7.5):32, (8.0):1
Occult blood	(−)	Test food	33	(−):31, (±):2	(−):30, (±):3
		Placebo	33	(−):30, (±):3	(−):29, (±):4

Note

The number of subjects with each result is shown.

**Table 4:** Diet survey (BDHQ)

Item		Ingested food	*n*	Preingestion	Post‐4W
Calories	kcal/day	Test food	33	1,766 ± 501	1,660 ± 437
		Placebo		1,736 ± 440	1,702 ± 418
Carbohydrate	g/day	Test food	33	219 ± 75	207 ± 64
		Placebo		217 ± 67	208 ± 56
Protein	g/day	Test food	33	73.8 ± 23.9	69.4 ± 21.2
		Placebo		72.5 ± 19.9	71.7 ± 21.7
Fat	g/day	Test food	33	59.0 ± 15.6	54.8 ± 14.3
		Placebo		58.1 ± 14.9	56.2 ± 16.6
Calcium	mg/day	Test food	33	631 ± 209	586 ± 185
		Placebo		598 ± 182	595 ± 190
Potassium	mg/day	Test food	33	2,779 ± 816	2,716 ± 788
		Placebo		2,850 ± 818	2,819 ± 894
Vitamin D	µg/day	Test food	33	14.6 ± 8.9	13.0 ± 6.8
		Placebo		13.6 ± 6.7	14.2 ± 7.6
Vitamin K	µg/day	Test food	33	334 ± 137	340 ± 149
		Placebo		335 ± 150	344 ± 156

Note

Values are mean ± *SD*.

There was a significant decrease in the DPD values with the consumption of the test food in the intragroup comparisons before and after the interventions (−0.81 ± 0.96, *p* = .011). Conversely, significant variation was not observed, even if the placebo food was ingested. In addition, intergroup comparisons showed that postintervention measurements (test food: 4.01 ± 1.11 nmol/mmol·Cr, placebo food: 4.80 ± 1.08 nmol/mmol·Cr, *p* = .006) as well as changes from preintervention to postintervention (test food: −0.81 ± 0.96 nmol/mmol·Cr, placebo food: +0.19 ± 0.71 nmol/mmol·Cr, *p* < .001) were significantly lower when the test food was consumed compared with the placebo food.

u‐NTx levels tended to decrease with consumption of the test food in intragroup comparisons before and after the interventions (test food: 42.9 ± 18.1 nmol BCE/mmol·Cr, placebo food: 53.5 ± 28.7 nmol BCE/mmol·Cr, *p* = .084). In the intergroup comparisons, the change from the preintervention level was significantly lower when the test food was consumed compared with when the placebo food was consumed (test food: −3.5 ± 15.0 nmol BCE/mmol·Cr, placebo food: +5.6 ± 20.0 nmol BCE/mmol·Cr, *p* = .047).

- Urinalysis

In the urinalysis, although items showing false positives (Pro, Bld) and slight positives (Ket) were occasionally observed, there was no significant difference between the groups, and the changes observed were not considered medically problematic.

- Diet survey

In the dietary survey by BDHQ, there were no significant differences in the various nutrients ingested between the groups.

## 4 DISCUSSION

The purpose of this study was to evaluate the effect of maltobionic acid intake on bone metabolism in Japanese women who were 40–69 years old and who were more than 1 year past menopause. In order to evaluate bone metabolism, bone density and bone metabolism markers are usually utilized. Because bone metabolism changes from day to day, the metabolic state is different even at the same bone density value (Miller, Hochberg, Wehren, Ross, & Wasnich, 2005). Therefore, an observation period of 6 months to 1 year is required to make bone density a dynamic marker. Conversely, as the bone metabolism markers quantitatively reflect bone metabolism at the time of measurement, they are useful to predict future bone loss and fracture risks and are essential examination items in the diagnosis of osteoporosis (Soen et al., 2013). Elevated bone resorption markers have been identified as fracture predictors in prospective studies (Ivaska, Gerdhem, Väänänen, Åkesson, & Obrant, 2010) and have also been reported to be risk factors for fractures independent of bone mineral density (Gerdhem et al., 2004).

In this study, DPD and u‐NTx were measured in urine specimens as bone resorption markers. As noninvasive markers, where blood collection is unnecessary, they are widely used in medical practice for the diagnosis of osteoporosis and confirmation of therapeutic effect (Eastell et al., 2000; Robins et al., 1991). In particular, DPD is mainly localized in bone and dental tissues, though dental tissues do not affect urinary excretion levels. Thus, bone can be regarded as the only tissue of origin for urinary DPD, which has been reported to be particularly useful as an indicator of bone resorption (Uebelhart et al., 1991). The postintervention values of DPD and u‐NTx did not differ in the placebo food group compared to the preintervention values; however, these values reduced significantly in the test food group. Among them, marked differences in DPDs were identified in the pre‐ and postintervention groups due to the consumption of the test food, as revealed by both intragroup comparisons (*p* = .011) and intergroup comparisons (*p* < .001). Maltobionic acid in the test food has been shown to inhibit the differentiation of osteoclasts in in vitro cell studies, and a significant reduction in bone resorption markers has also been observed in in vivo animal studies in menopausal model mice (in preparation). Further, another study determined that in an in situ ligated rat jejunal loop absorption test, the Ca component of the MBCa was selectively absorbed, whereas the maltobionic acid component was confirmed to also be slightly absorbed in the small intestine (Suehiro et al., 2017). From this, it is hypothesized that the release of DPD and u‐NTx is lowered by suppressing differentiation and bone resorption of the osteoclast by maltobionic acid being absorbed in the intestine.

In addition, an increase in bone density has been reported with a decrease in bone resorption markers in clinical trial cases with drugs for the treatment of osteoporosis (Chesnut et al., 1995; Marttunen et al., 1999). Maltobionic acid has also been shown to maintain or increase bone mineral density in a 24‐week intervention study in postmenopausal women (Fukami et al., 2019), which may originate from the inhibition of bone resorption. Furthermore, in an animal study in rats, it was confirmed that maltobionic acid maintained the solubilization state of calcium throughout the intestine, thereby increasing the calcium retention rate (Suehiro et al., 2019). It has been reported that the bioavailability of calcium is closely related to bone density and bone metabolism (Cashman, 2002; Weaver, 1992). Maltobionic acid may have further contributed to the improvement of bone metabolism by enhancing the bioavailability of calcium.

Finally, the efficacy outcomes in this study were limited to the markers of bone resorption. For further assessment of the effects on bone turnover, markers of bone formation (e.g., OCs, BAPs) and bone matrix‐related markers (e.g., ucOC, pentosidine) must also be included in the outcomes and analyzed comprehensively (Eastell et al., 2018; Eastell & Szulc, 2017). Additionally, quantification of serum parathyroid hormone and calcitonin, which are blood markers, is necessary to evaluate the increase in calcium bioavailability by maltobionic acid and their effects on markers of bone metabolism. Further research is necessary to clarify the mechanism underlying bone metabolism improvement effect by the intake of maltobionic acid.

In conclusion, continuous intake of maltobionic acid in healthy Japanese adult women was found to suppress bone resorption and improve bone metabolism, which may contribute to the prevention of osteoporosis.

## CONFLICT OF INTEREST

Daiki Suehiro and Ken Fukami are employees of San‐ei Sucrochemical Co., Ltd. San‐ei Sucrochemical Co., Ltd. is the supplier of a food product containing maltobionic acid. Ayaka Nishio, Jyunya Kawai, and Motoko Ohnishi have no conflict of interest to declare.

## AUTHOR CONTRIBUTIONS

Daiki Suehiro and Motoko Ohnishi designed the research protocol. Daiki Suehiro and Ken Fukami provided the test and placebo foods. Daiki Suehiro wrote the manuscript. Ken Fukami and Motoko Ohnishi reviewed and edited the manuscript. Motoko Ohnishi had primary responsibility for the final content. Ayaka Nishio and Jyunya Kawai participated in conducting the analyses. All authors read and approved the final version of the manuscript.

## ETHICAL APPROVAL

This study was approved by the Chubu University Certified Review Board on September 21, 2018 (no. 300019‐2).

## INFORMED CONSENT

Written informed consent was obtained from all study participants.
